# Review and Critical Appraisal of Clinical Practice Guidelines of Modalities Used in the Diagnosis of Celiac Disease

**DOI:** 10.1093/jcag/gwad005

**Published:** 2023-04-01

**Authors:** Kennedy Graham, Dominica Gidrewicz, Justine M Turner, Donald R Duerksen, Maria Ines Pinto-Sanchez

**Affiliations:** Department of Medicine, Farncombe Family Digestive Health Institute, McMaster University, Hamilton, Ontario, Canada; Department of Pediatrics, Alberta Children’s Hospital Research Institute, University of Calgary, Calgary, Alberta, Canada; Department of Pediatrics, University of Alberta, Edmonton, Alberta, Canada; Department of Internal Medicine, Section of Gastroenterology, University of Manitoba, Winnipeg, Manitoba, Canada; Department of Medicine, Farncombe Family Digestive Health Institute, McMaster University, Hamilton, Ontario, Canada

## Abstract

**Background:**

There is controversy over the recommendations for specific serological strategies implemented and the need for a biopsy to confirm celiac disease (CeD). We reviewed and appraised the current clinical practice guidelines (CPGs) to assess the quality and reliability of recommendations for CeD diagnosis in pediatric and adult populations.

**Methods:**

We searched databases, including MEDLINE, EMBASE, Web of Science, and CINAHL, between December 2010 and January 2021 for CPGs. Four independent reviewers extracted data. Appraisal of Guidelines Research and Evaluation (AGREE II) criteria were applied by two reviewers, and a standardized score was calculated for each of the six domains. A cut-off of 60% was used to identify high-quality guidelines.

**Results:**

A total of 654 records were identified, 10 of which were eligible for data extraction. Both adult and pediatric CPGs averaged above 70% for the domains of ‘scope and purpose’ and ‘clarity and presentation’. For ‘stakeholder involvement’, the mean adult and pediatric CPG scores were below the cut-off. Only one adult-focused guideline exceeded the cut-off for the ‘rigour of development’ domain. ‘Applicability’ scores were most alarming, with adult CPGs averaging 21% and pediatric CPGs averaging 23%.

**Conclusion:**

Our review and appraisal of the CPGs for the diagnosis of CeD highlight significant discrepancies in clinical recommendations and some concerns regarding methodological rigour, particularly in stakeholder engagement, rigour, and applicability. Creating a Canadian guideline of high methodological quality that overcomes these weaknesses is critical to optimize patient care and ensuring accurate diagnoses in CeD.

## Teaser

Accurate tests are important for the diagnosis of celiac disease (CeD), a reaction of the immune system after eating gluten, the protein found in wheat, rye and barley. There is controversy over the recommendations on which test is more effective for the diagnosis of CeD. We reviewed the current guidelines (CPGs) with recommendations on the most appropriate test for diagnosis of CeD and rated the guidelines using a valid tool to assess the scientific content of recommendations for CeD diagnosis in pediatric and adult populations. We searched several databases to find these guidelines published in medical journals between December 2010 and January 2021. Two reviewers rated the scientific quality of the guidelines using the Guidelines Research and Evaluation (AGREE II). Based on previous studies using this tool, we considered that a score over 60% was a high-quality guideline. We found inconsistencies in recommendations on how to diagnose CeD, and some concerns regarding methodological rigour between guidelines. Inconsistent recommendations can confuse providers and funders, leading to a delay in diagnosis and unnecessary diagnostic procedures. We hope future guideline developers will use the strengths and weaknesses identified in this review to help physicians, government institutions and patients.

## Graphical Abstract

**Graphical Abstract:**
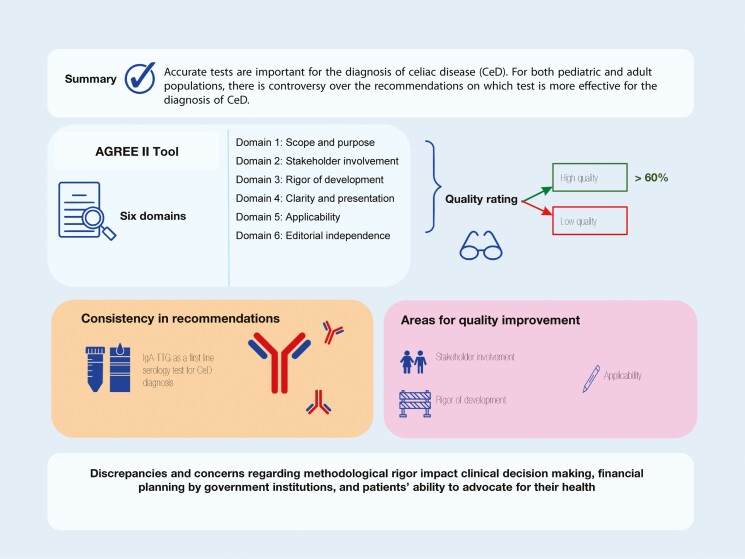

## BACKGROUND

Celiac disease (CeD) is caused by an immune-mediated reactio sted gluten and related prolamins in genetically susceptible individuals (1). In Canada, about 1% of people are affected by CeD (2). CeD can manifest clinically with both gastrointestinal (GI) and extraintestinal symptoms, although many patients are asymptomatic at diagnosis (1–4). Accurate and timely diagnosis, followed by appropriate treatment, is instrumental to improving both GI and extraintestinal symptoms in patients with CeD, preventing clinical deterioration and complications in the longer term (5). The traditional diagnosis of CeD is based on the presence of specific antibodies and confirmation of enteropathy in duodenal biopsies (6). However, the specific serological strategies implemented and the utilization of a biopsy to confirm CeD diagnosis has been controversial.

Guidelines educate and reinforce the importance of the most evidence-based diagnostic tests for physicians and government bodies that fund testing services. Several clinical practice guidelines (CPGs) from various countries and GI organizations provide recommendations regarding the diagnosis of CeD. Furthermore, there is increasing concern about the lack of standardization and variability in advice between societal guidelines. For example, the European Society for Paediatric Gastroenterology Hepatology and Nutrition (ESPGHAN) (7, 8) recommends a nonbiopsy approach for the diagnosis in the pediatric population meeting specific criteria. Still, other guidelines do not adopt this recommendation. Similar discrepancies are seen in the recommendations pertaining to various diagnostic strategies using CeD-specific serology or a combination of tests.

Inconsistencies in the recommendations from different guidelines can confuse providers and funders, leading to a delay in diagnosis and unnecessary diagnostic procedures. Therefore, we performed a review and appraisal of the current guidelines, using the Appraisal of Guidelines Research and Evaluation (AGREE II) criteria (9, 10), to assess the quality and reliability of recommendations for the diagnosis of CeD in both pediatric and adult populations.

## METHODS

### Literature Search

A systematic search for guidelines published from December 2010 to January 2021 was conducted, using the following keywords: ‘diagnosis’, ‘guidelines’, ‘practice guideline’, ‘celiac disease’, ‘guidelines’ and ‘celiac disease guideline’ in the following databases: MEDLINE, EMBASE, CENTRAL, Web of Science, and CINAHL. The research strategy used was the following: (Celiac disease OR celiac sprue OR gluten sensitive enteropathy) AND (diagnosis/) AND (guidelines.mp OR practice guideline/). We included papers identified as guidelines on the diagnosis of celiac disease published in the last 10 years, independently of the methodology used for the guideline development. There were no restrictions by country or language; translations were obtained by the reviewers or a third person familiar with the language. Previous versions of CPGs were included to expose changes in recommendations.

For this appraisal, CPGs were defined as statements that include recommendations intended to optimize patient care, informed by a systematic review of evidence. Reviews of the literature not including recommendation statements, or expert reviews (not systematic reviews) were not considered CPGs and therefore, excluded from the analysis. In addition, CPGs that did not address the question of the modality of diagnosis in CeD were excluded from the analysis. For the selection of the resulting CPGs, four authors (MIPS, JT, DG, DD) conducted an individual title and abstract review to assess inclusion criteria. The full-text documents of preselected guidelines were then reviewed to verify their eligibility after exclusion criteria were applied, such as design different than a guideline i.e., expert reviews, systematic reviews, or editorials. Guidelines selected under these conditions were screened using the selection criteria by a consensus of two authors.

For each guideline, the date of publication, journal title, population, number of centers, guideline language and country of origin, guideline developers, funding source, and recommendations were extracted by two independent reviewers. Subsequently, Appraisal of Guidelines for Research & Evaluation (AGREE) II criteria were applied by two independent raters. The AGREE II instrument was used for guideline appraisal to assess the methodological quality of the CPG (11). AGREE has been updated and refined, proven valid and reliable, and is internationally accepted for the evaluation of practice guidelines (12, 13)^.^ It contains 23 key items in the following six domains: scope and purpose, stakeholder involvement, the rigour of development, clarity of presentation, applicability, and editorial independence. Before the rating process was begun, the contents of each item of the AGREE II checklist were discussed. In the case of disagreement between the two reviewers, a third reviewer was involved in a decision. AGREE II checklist is attached as Supplementary Data.

Each domain of the AGREE II tool was calculated by summing up the scores of the individual items in a domain and then standardizing as follows: (score obtained–minimum possible score)/(maximum possible score–minimum possible score). The maximum score for each domain was the number of questions multiplied by the number of reviewers, multiplied by the number of scores of seven (strongly agree). The minimum score was the number of questions multiplied by the number of reviewers, multiplied by the number of scores of one (strongly disagree). Therefore, the minimum standard score for each domain was 0%, and the maximum was 100%. Based on previous work and the consensus of experts, we considered a minimum of 60% on each AGREE domain as a cut-off for a good quality guideline (14–16).

A descriptive statistical analysis was performed for each domain. Descriptive measures used were average, standard deviation, minimum and maximum. The individual results of each domain are presented as a percentage. The agreement between the evaluations of the two reviewers for each guideline was determined by the intraclass correlation coefficient (ICC) with 95% CI. Analyzes were performed using SPSS (Statistical Package for the Social Sciences, Chicago, IL, United States), version 21.

## RESULTS

**Figure 1.**
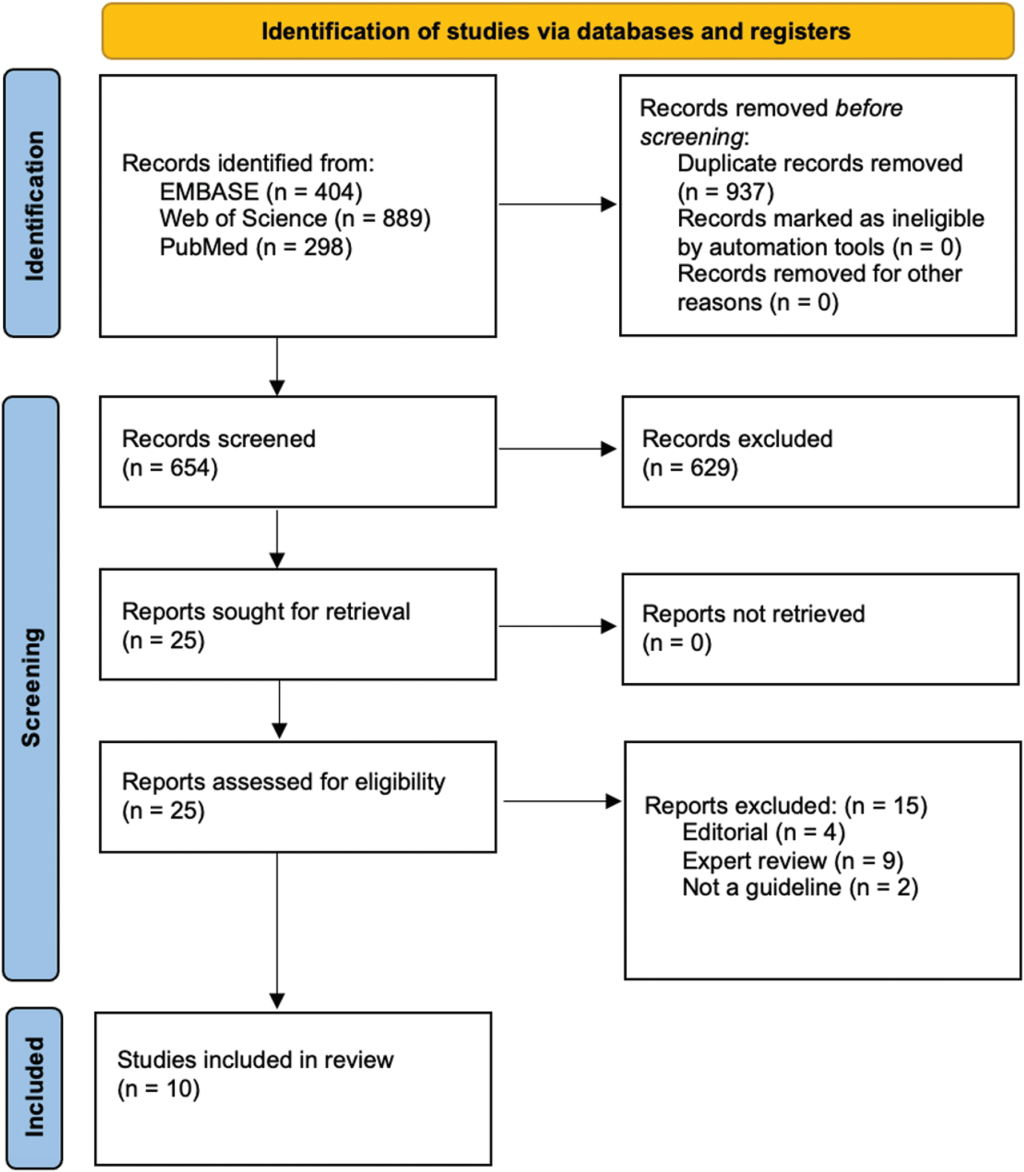
PRISMA flow chart for literature search.

The literature search retrieved 404 records in the EMBASE database, 889 in Web of Science, 298 in PubMed. When duplicates were removed, a total of 654 records were included. Following the title and abstract search, 25 texts met the inclusion criteria. Of the full texts, only 10 met the inclusion and exclusion criteria and were included in the analysis (Figure 1). Most guidelines were authored by a professional organization or government agency and developed by physicians and individuals working on a CeD clinical management team, including pathologists, endoscopists, immunologists, and dieticians. As shown in Supplementary Table 1, the most common reason for exclusion was that the publication was a paper and not a guideline (i.e., expert review or editorial).

There was one American clinical practice guideline (ACG) (17), three from Britain (BSPGHAN (18), BSG (19) and NICE (20)), one from Mexico (Asociacion Mexicana de Gastroenterologia (21)), and three from Europe (ESPGHAN (7, 8), ESSCD (22)) two of which were presented by ESPGHAN, written in 2012 (7) and updated in 2020 (8). Finally, two guidelines were from the World Gastroenterology Organization (WGO (23, 24)), written in 2013 (23) and updated in 2017 (24).

### Recommendations for the Adult Population

#### Serological Testing

ESSCD and BSG recommend that diagnostic serologic testing in adult populations should be done while patients are on a gluten-containing diet (19, 22). Seven of the ten CPGs recommend IgA TTG as the single preferred test for CeD detection at any age and indicate that total IgA levels need to be assessed concurrently (7, 8, 17, 18, 20, 22, 24). No guidelines suggested a test other than IgA TTG to be the first-line test. Several CPGs recommend that if IgA TTG is weakly positive, IgA EMA should be used concurrently with IgA levels (7, 13, 17, 20, 21, 24). Furthermore, for adults who are IgA deficient, the guidelines recommend IgG DGP (7, 8, 17, 19–24), IgG TTG (17–22, 24), or IgG EMA (7, 8, 18, 20, 24) (see Table 1).

**Table 1.** Summary of included guidelines for the diagnosis of CeD in adult and pediatric populations

Study, year, reference	Society	Population	Serology strategy	Duodenal biopsy	Nonbiopsy approach	HLA	WCE
Rubio-Tapia 2013 (17)	American College of Gastroenterology (ACG)	Pediatric/adult	IgA TTG + total IgA or IgG DGP or IgG TTG. <2 years old, combine IgA TTG with IgG and IgA DGP	Small bowel biopsy is critical in diagnosis. At least 4 biopsies of distal duodenum; 1-2 from the bulb	Not mentioned	Should only be used in adults to rule out or exclude CeD	In patients with complicated CeD, or those who are unable or unwilling to undergo upper endoscopy with biopsy
Bai 2013 (23)	World Gastroenterology Organization (WGO)	Pediatric/adult	IgA TTG or IgA EMA. <3 years old, IgA DGP or IgG DGP. If IgA deficient, IgG DGP	Duodenal biopsy is the gold standard. 3-4 biopsies from duodenum; at least 1 from bulb	Favoured in low resource setting	Should only be used in adults to rule out or exclude CeD	Not addressed
Husby 2020 (8)	European Society Paediatric Gastroenterology, Hepatology and Nutrition (ESPGHAN)	Pediatric/adult	IgA TTG + total IgA If IgA deficient, IgG DGP or IgG EMA or IgG TTG	If serologic criteria are not met, duodenal biopsy required. 4 biopsies from the distal duodenum, at least 1 from the bulb	Favoured when TTG test shows PPV>95% and in asymptomatic children	Not required in patients with TTG+, if they qualify for CeD diagnosis with biopsies, or have TTG > 10 ULN and EMA+	Not addressed
Husby 2012 (7)	European Society Paediatric Gastroenterology, Hepatology and Nutrition (ESPGHAN)	Pediatric/adult	IgA TTG or IgA EMA + total IgA If IgA deficient, IgG TTG or IgG DGP or IgG EMA	In asymptomatic patients or those not meeting serological criteria, duodenal biopsy is required. 4 biopsies from the distal duodenum, at least 1 from the bulb	Favoured when TTG test shows PPV>95% and in asymptomatic children	In children for nonbiopsy diagnosis, screening in at-risk groups, and in patients with mild infiltrative changes on small bowel biopsies but negative CeD-specific antibodies	Not addressed
Murch 2013 (18)	British Society of Pediatric Gastroenterology, Hepatology and Nutrition (BSPGHAN)	Pediatric/adult	IgA TTG + total IgA If IgA deficient, IgG TTG or IgG EMA	If TTG raised, but less than 10 × ULN, duodenal biopsy is required; 4 biopsies from distal duodenum and 1-2 from the bulb	Favoured; if EMA+ and patient DQ2 or DQ8. If EMA antibody testing is not locally available, a second strongly positive TTG antibody	In asymptomatic children with associated conditions and negative serology	Not addressed
Ludvigsson 2014 (19)	British Society of Gastroenterology (BSG)	Adult	IgA TTG If IgA deficient, use IgG DGP or IgG TTG	Biopsy remains essential for diagnosis of adult CeD. At least 4 duodenal biopsies, 1 from the bulb	Against; IgA deficiency may lead to false negatives in serological testing, not all commercial IgA-TG2 kits are reliable	Self-treated on a GFD without appropriate testing for CeD; used to rule out CeD and minimize future testing; used in high-risk individuals with CeD (i.e., first-degree relatives)	In adults who are unable or unwilling to have an endoscopy. Capsule endoscopy may have a supportive role
Al-Toma 2019 (22)	European Society for the Study of Celiac Disease (ESSCD)	Adult	All serologic testing should be done on a gluten-containing diet; IgA TTG preferred single test If IgA deficient, use IgG DGP or IgG TTG	Recommended to confirm the diagnosis of CeD. 4 duodenal biopsies, two from the bulb	No recommendation; need more data before eliminating biopsy from the diagnostic process	Rule out CeD in the following populations: Marsh 1-2 histology in seronegative patients; GFD initiated before CeD testing; Discrepancy between serology and histology	In adults who are unable or unwilling to have an endoscopy. Also important for detecting complications with CeD
Remes-Troche 2018 (21)	Asociacion Mexicana de Gastroenterologia	Adult	IgA TTG + IgG DGP or IgA EMA	Recommended for diagnosis. 6 duodenal biopsies (4 from D2-3, 2 from D1)	No recommendation; recognizes ESPGHAN recommendation for pediatric cases that could be used in adults, but not widely implemented	Rule out CeD in the following populations: seronegative cases, those with discrepant results in serology and biopsy, and those on GFD without investigations for CeD	Not addressed
Bai 2017 (24)	World Gastroenterology Organization (WGO)	Adult	IgA TTG or IgA EMA + total IgA; if IgA deficient, IgG DGP, IgG TTG, or IgG EMA. Asymptomatic patients with positive serology should be retested after 3 months of gluten-containing diet before endoscopy	Symptomatic patients with a positive serological test or a titer just below the cut-off should have multiple duodenal biopsies to confirm or exclude the diagnosis of CeD. 4-6 biopsies from second part of duodenum, biopsy should be taken from bulb but did not specify how many	Biopsies may be omitted in certain situations; must discuss pros and cons with expert physician	Rule out CeD in the following populations: Seronegative celiac, those with discrepant results, those with first-degree family members, and those with other autoimmune conditions i.e., autoimmune thyroiditis.	Not addressed
NICE 2015 (20)	National Institute for Health and Care Excellence (NICE)	Adult	IgA TTG + total IgA; use IgA EMA if IgG TTG is weakly positive. If IgA deficient, use IgG EMA, IgG DGP, or IgG TTG	Biopsy should be performed to confirm or exclude CeD diagnosis; number of biopsies not described	Against; Serology is imperfect and there is great variation in the assays used and the inter-test reliability within each laboratory, likelihood of a false-positive diagnosis may be increased	Consider HLA testing only in the diagnosis of CeD in specialist settings; for example, in children who are not having a biopsy, or in individuals who already have limited gluten ingestion and choose not to have a gluten challenge	Not addressed

#### Duodenal Biopsy

The majority of guidelines recommend that confirmatory biopsies be taken in adult populations. Five guidelines (17, 19, 20, 22, 24) determined that a biopsy is essential for diagnosis, even with positive serology. ESSCD recommends that duodenal biopsies should be taken when CeD is suspected, despite a normal endoscopic view and negative serology (22). WGO specifically recommends that symptomatic patients with positive serological tests or titers just below the cut-off should be referred for endoscopy with multiple duodenal biopsies to confirm or exclude a diagnosis of CeD (24). However, WGO recommends that asymptomatic patients with positive serological tests should be retested after 3 months (while consuming a gluten-containing diet) to confirm seropositivity before referral for endoscopy (24).

Eight guidelines recommend that at least 4 duodenal biopsies should be taken in adult patients (7, 8, 17–19, 22–24). In contrast, the Asociacion Mexicana de Gastroenterologia recommends taking six duodenal biopsies (21). Moreover, six guidelines recommend that at least one biopsy is taken from the duodenal bulb (7, 8, 17–19), whereas two CPGs recommend that at least two biopsies are taken from the bulb (21, 22).

NICE and BSG recommend against a nonbiopsy approach for the following reasons: Not all IgA TTG testing kits are reliable, and there is a high potential for false negatives and reduced test sensitivity because 2% of patients with CeD are IgA deficient (19, 20). Three guidelines would consider a nonbiopsy approach under the following conditions: (a) in children when specific criteria are met, including a TTG value with a PPV>95% (8) and the patient is symptomatic (7) and (b) in adults in a low resource setting (23). EMA is recommended to support the diagnosis (7, 8, 18). One guideline states the decision to utilize a nonbiopsy approach must be made on a case-by-case basis after a discussion of the pros and cons with an expert physician (24). The remaining guidelines state more data are required in adults before they can endorse a nonbiopsy recommendation (21, 22).

#### HLA DQ2/DQ8 Testing

Generally, guidelines recommend that HLA testing could be used in adults to exclude CeD in the following populations: (a) high-risk individuals (7, 19), (b) individuals with other autoimmune conditions (23), (c) seronegative patients with Marsh 1-2 histology (7, 21, 22), (d) patients who have not had serological testing before adopting a GFD (19, 21, 22), (e) patients with discrepant CeD specific serology and histology results (21, 22, 24) and (f) first-degree family members (19, 24). There is a lack of consensus on absolute indications.

#### Screening for CeD in People Undergoing Endoscopy

Most CPGs for adult CeD did not address whether individuals undergoing endoscopy should be screened for CeD. WGO and BSG guidelines recommend that biopsies should be obtained when any characteristic endoscopic features are observed (19, 24).

#### Wireless Capsule Endoscopy (WCE)

Three guidelines recommend WCE for adults who are unwilling to have an endoscopy (17, 19, 22). In addition, ESSCD recommends WCE for detecting complications (22), and ACG recommends WCE for evaluating the small bowel mucosa in patients with complicated CeD (17).

### Recommendations in the Pediatric Population

#### Serological Testing

There are variations in the recommendations for serological testing in the pediatric population. Like adult CPGs, all pediatric CPGs agree that IgA TTG should be the single preferred test for the detection of CeD (7, 8, 17, 18, 23). Four guidelines recommend measuring IgA levels first to determine the most appropriate serological test (7, 8, 17, 18). WGO and ACG recommend IgA TTG for individuals over age 2 and IgA DGP if a patient is under 3 years old (17, 23). Several other studies recommend IgA TTG regardless of age provided the patient has a normal total IgA (7, 8, 18). If a pediatric patient has IgA deficiency, there is consensus on the use of IgG-based testing; IgG TTG (7, 8, 17, 18), IgG DGP (7, 8, 17, 23) or IgG EMA (7, 8) are recommended (see Table 1).

#### Duodenal Biopsy

Four guidelines favour a nonbiopsy approach (7, 8, 18, 23), and one did not mention a nonbiopsy approach in their recommendations (17). The reasons for using a nonbiopsy approach include lack of resources in certain settings (23) and when a TTG test has a PPV > 95% (8). ESPGHAN is the only guideline that includes asymptomatic pediatric patients, recommending a nonbiopsy approach even for these patients if they meet the serologic criteria (8). When a duodenal biopsy is deemed necessary, all guidelines recommend that physicians take at least 1 biopsy from the bulb and at least 4 biopsies from the distal duodenum (7, 8, 17, 18, 23).

#### HLA DQ2/DQ8 Testing

There is a consensus that HLA DQ2/DQ8 should not be used routinely at an initial diagnosis of CeD in pediatric patients but rather used as a rule-out test with specific criteria to support exclusion of CeD in ambiguous cases (7, 8, 17, 18, 23). Specifically, the recent ESPGHAN guideline (unlike the earlier version) now recommends that HLA testing is not required in patients with positive TTG if they qualify for the CeD diagnosis with serology (nonbiopsy) (8). One guideline recommends that HLA be considered in asymptomatic children with an associated condition and negative serology (18).

#### Screening for CeD in People Undergoing Endoscopy

Only ESPGHAN addressed screening for CeD in pediatric patients undergoing endoscopy, recommending that antibody testing (IgA TTG for individuals >2 years old, and IgG DGP for children <2 years old) or HLA DQ2/DQ8 be used (7).

#### Wireless Capsule Endoscopy (WCE)

One guideline recommended WCE for patients who are unwilling or unable to undergo upper endoscopy with biopsy and those with complicated CeD (17). In other pediatric guidelines, WCE was not addressed. Further research on this topic is necessary to determine an appropriate recommendation.

### Guidelines Appraisal: AGREE II Instrument

The overall ICC value among reviewers was very good for both adult guidelines (0.966% CI 0.938 to 0.981) and pediatric guidelines (0.973% CI 0.915 to 0.989). Tables 2 and 3 show the mean scores of each domain.

**Table 2.** Score domains and overall assessment of CPGs providing recommendations for adult CeD according to AGREE II instrument

CPG, year and reference	Scope and purpose (%)	Stakeholder involvement (%)	Rigour of development (%)	Clarity and presentation (%)	Applicability (%)	Editorial independence (%)
Ludvigsson 2014 (19)	81	67	42	94	2	83
Al-Toma 2019 (22)	81	47	42	78	4	0
Remes-Troche 2018 (21)	56	72	44	56	13	67
Bai 2017 (24)	69	56	13	72	4	46
NICE 2015 (20)	100	100	81	94	71	79
Rubio-Tapia 2013 (17)	81	36	0	94	23	83
Bai 2013 (23)	11	31	0	25	8	0
Husby 2020 (8)	97	50	59	97	23	83
Murch 2013 (18)	72	47	0	100	21	0
Husby 2012 (7)	94	44	56	97	42	83
Mean scores for each domain	74	55	34	81	21	53

**Table 3.** Score domains and overall assessment of CPGs providing recommendations to paediatric CeD according to AGREE II instrument.

CPG, year and reference	Scope and purpose	Stakeholder involvement	Rigour of development	Clarity and presentation	Applicability	Editorial independence
Rubio-Tapia 2013 (17)	81	36	0	94	23	83
Bai 2013 (23)	11	31	0	25	8	0
Husby 2020 (8)	97	50	59	97	23	83
Murch 2013 (18)	72	47	0	100	21	0
Husby 2012 (7)	94	44	56	97	42	83
Mean scores for each domain	71	42	23	83	23	50

Considering individual instrument domains, both adult and pediatric CPGs scored above 70% for ‘scope and purpose’ and for ‘clarity and presentation’ (see Tables 2 and 3). The average score for the ‘scope and purpose’ domain was 74%; all but two of the guidelines exceeded the cut-off of 60% (7, 8, 17–20, 22, 24). The ‘clarity of presentation’ domain deals with key recommendations and whether they are easily identifiable, specific, and unambiguous (25). It also determines whether different options for the management of CeD are presented (25). For adult and pediatric CPGs, ‘clarity of presentation’ was the domain with the highest average score. All but two of the guidelines exceeded the cut-off of 60% in this domain as well (7, 8, 17–20, 22, 24).

On average, the guidelines fell below the cut-off for ‘stakeholder involvement’, the domain which assesses whether the guideline development group included individuals from all relevant professional groups, sought the views and preferences of the target population and clearly defined the target users (25). The average score for this domain for adult CPGs was 55% (range 31% to 100%). Only three guidelines exceeded the cut-off of 60% (19–21). The average score for pediatric guidelines was 42% (range 31% to 50%), with no guidelines exceeding the cut-off. Those that did not meet the cut-off lacked an evaluation of patient preferences and viewpoints (7) and did not include all relevant providers in recommendation development (8, 17, 18, 23).

The ‘rigour of development’ domain evaluates the methods used in formulating recommendations through a systematic collection of data and summarizing the body of evidence (10, 12). It ensures the link between recommendations and supporting evidence and the presence of methods to update guidelines (11, 12, 25). The average score for adult guidelines for this domain was 34% (range 0% to 81%). Only one guideline exceeded the cut-off (20). Most pediatric CPGs lacked in the ‘rigour of development’ domain. None of the guidelines scored above the cut-off, although ESPGHAN came close, scoring 59% (8). Four CPGs did not describe a systematic approach or search methods (7, 17, 18, 23), and 3 CPGs did not describe benefits and harms (19, 22, 24). In addition, both versions of the ESPGHAN guidelines did not have an external review or detail a process to update the recommendations (7, 8).

The applicability scores for CPGs were alarming. The mean score for adult guidelines in the ‘applicability’ domain was 21% (range 2% to 71%). Only 1/10 guidelines, NICE, scored above the cut-off at 71% (20). Similarly, the average score for pediatric guidelines in this domain was 23% (range 25% to 100%). 4/5 pediatric guidelines scored below 25% (8, 17, 18, 23)^,^. The ‘applicability’ domain scores highlighted a lack of awareness of costs, institutional facilitators and barriers, and resource implications when implementing several of the guidelines (7, 8, 18, 23). With low applicability, CPGs will lack adherence or will be poorly utilized.

Finally, the mean score for the ‘editorial independence’ domain for adult guidelines was 53% (range 0% to 83%), and 6/10 adult CPGs received a score above the cut-off in this domain (7, 8, 17, 19–21). The mean score for this domain for pediatric guidelines was 50% (range 0% to 83%), and 3/5 guidelines scored above the cut-off (7, 8, 17). Those that did not receive a score above the cut-off lacked funding or a conflict of interest statement.

## DISCUSSION

Our review and appraisal of the CPGs for the diagnosis of CeD published since 2012 showed major discrepancies in clinical recommendations and some concerns regarding methodological rigour, particularly in stakeholder engagement, rigour and applicability domains. In general, clarity was less of a concern among all the guidelines. The guidelines that scored lower in the clarity domain faced ambiguity in some key recommendations (21, 23, 24) and lacked a summary tool that allowed readers to access the recommendations in a clear and concise manner (23, 24).

One concern is that CPGs did not evaluate patient preferences or viewpoints (7, 19, 21, 23, 24), particularly given the high prevalence of CeD and well-established patient organizations. Others did not pilot the guideline created, an essential step for ensuring guideline uptake (21, 22, 24). Moreover, CPG development should involve individuals from all relevant areas; however, we found one CPG (23) only included physicians, and others (8, 18, 22) targeted only GI providers. These guidelines do not establish the characteristics of their target populations and do not recognize each stakeholder’s role in developing the guideline. Lack of patient involvement could also mark a significant flaw in the knowledge translation of a guideline, including the importance of maintaining a gluten-containing diet in the accuracy of serologic testing. In addition, easily understandable guidelines empower patients to make more informed healthcare choices and to consider their personal needs and preferences in selecting the best options (26). Overall, this review points to the possible reasons why existing CeD guidelines do not often translate well in practice (27, 28).

Moreover, clinicians have the freedom to debate whether or not to follow a CPG recommendation. As such, it is crucial for CPGs to accurately and conscientiously synthesize evidence to provide clarity and ease of implementation for practicing physicians. This makes the ‘rigour of development’ domain fundamental. The most common weaknesses related to the rigour of development include the following: Unclear search methodology, the harms and benefits of the CPG were not considered (19, 22), there was no update plan provided (8, 21, 22), and there was no external review performed (7, 8, 21, 22). Differences in the rigour of development may explain the differences in recommendations on modalities of diagnosis in CeD, observed across CPGs.

The ‘applicability’ domain had the lowest average score. Resource implications were not considered in all but one of the guidelines, and cost-effectiveness for each recommendation was often missed (7, 8, 19, 22, 23). This is problematic because such factors likely influence a patient’s or physician’s decision to follow recommendations, and again this can limit the translatability of the guideline. Further, three CPGs (21, 22, 24) did not consider the broad facilitators and barriers to recommendation implementation. Finally, several CPGs also did not include a strategy for following up with the patient and monitoring to evaluate the impact of the recommendations (19, 21, 22, 24). As such, only one British guideline, NICE, scored above the 60% cut-off (20). This highlights an area for improvement related to the analysis of facilitators and barriers and the tools to overcome the obstacles their recommendations may have.

Editorial independence, an essential criterion for CPGs, was identified in six of the CPGs (7, 8, 17–19, 21). In contrast, the funding body (22, 24) or conflict of interest (20, 22, 23) was not reported in three CPGs (20), however, conflict of interest for the NICE guideline committee members are available in their website under history tab. Overall, many CPGs did not describe how each author had been influenced by their funding source, bringing the motivations behind recommendations into question.

Despite developing several guidelines, there remains controversy around certain aspects of diagnosis in pediatric and adult CeD. This study highlights areas of improvement for the existing CeD CPGs, namely in the domains of ‘stakeholder involvement’, ‘rigour of development’ and ‘applicability’. By identifying areas for improvement, this review encourages future guideline developers to focus on the target population and create summaries, algorithms, and electronic tools to overcome barriers and limitations for health professionals (9, 29).

We identified most CPGs published were from US and Europe, which highlights the need for local guidelines. For instance, Canada lacks its rigourous guideline for the diagnosis of CeD and recommendations coming from US guidelines may not apply to Canadians. Among the different guidelines, there is one key consistency that IgA TTG is the preferred first-line serology test in CeD diagnosis. In Canada, and more specifically in the province of Ontario, the TTG test has not been covered by the public health system until 2021, despite consistent recommendations by CPGs and being standard clinical practice around the world (30). The cost of the test is a barrier to diagnosis for many individuals, especially those who need it most. This highlights the importance of having local CPGs for healthcare systems, government bodies, or private insurers, which serve as a framework for clinical decisions and support best practices in the selection of diagnostic tests in CeD.

Our review of the CeD guidelines was a rigourous process. We attempted to include as many databases as possible, without language restriction, to decrease the chances of bias; however, we acknowledge some limitations related to our review. The AGREE II instrument is a subjective tool that allows various interpretations. We attempted to decrease this bias by having more than two assessors for each CPG, as recommended by the AGREE II developers (12). Furthermore, the two reviewers performed the AGREE II assessment independently and blinded. Regardless, there was a high level of agreement amongst reviewers, generating increased confidence in our results.

In conclusion, the discrepancies in current recommendations for the diagnosis of CeD between CPGs are concerning. The growing gap between guideline developers and users has been described as a ‘crisis of evidence-based medicine’ (29), which can lower the quality of care in CeD and hinder successful patient outcomes. Given that this crisis has been observed for the diagnosis of CeD based on current guidelines, this study provides some guidance around process improvements necessary for new CeD guidelines going forward (9, 29). Specifically, this review and appraisal of current CPGs highlight the need to develop evidence-based guidelines for the diagnosis of CeD that carefully considers patient preferences or viewpoints and utilizes a summary tool for ease of clinical implementation. We hope future CPGs developers will use the strengths and weaknesses identified in this review to help physicians in clinical decision-making, government institutions in financial planning for healthcare services, and patients advocating for their health.

## Supplementary Material

gwad005_suppl_Supplementary_Material

## Data Availability

No new data were generated or analyzed in support of this article. CPGs included in the review can be found through the following databases: MEDLINE, EMBASE, Web of Science, and CINAHL, or in the references section of this article
