# Supplementary material for: Review and Critical Appraisal of Clinical Practice Guidelines of Modalities Used in the Diagnosis of Celiac Disease
Source: J Can Assoc Gastroenterol. 2023 Apr 1;6(3):106–14. doi: 10.1093/jcag/gwad005 (PMC10235591; doi:10.1093/jcag/gwad005)
Supplement: gwad005_suppl_Supplementary_Material [file gwad005_suppl_supplementary_material.docx]

SUPPLEMENTAL TABLE 1: Excluded guidelines; reasons for exclusion.

| Excluded guideline | Reason for exclusion |
| --- | --- |
| Rubio-Tapia 2019 | Editorial. Not a CPG. |
| Husby 2018 | Expert review. Not a CPG. |
| Robert ME 2018 | Expert review- statement. Not a CPG. |
| Bibbins Domingo 2017 | Expert systematic review- Task Force on the need for screening in asymptomatic celiac. Not addressing question of interest. |
| Lazebnik LB 2015 | Expert review. Not a CPG. |
| Downey L 2015 | Expert review- Summary of NICE guideline. Not a CPG. |
| Nadhem 2015 | Not a CPG |
| Hill P 2015 | Editorial on BSG guidelines. Not a CPG. |
| Suomalaisen 2015 | Editorial. Not a CPG. |
| Kelly CP 2015 | Expert review. Not a CPG. |
| Trovato CM 2015 | Not a CPG. |
| Hill ID 2016 | Expert review. Not a CPG. |
| Jones HJ 2010 | Editorial. Not a CPG. |
| Leonnard M 2020 | Expert review. Not a CPG |
| Cheng FW 2020 | Expert review. Not a CPG. |

CPG=Clinical practice guideline

References

1- Rubio Tapia A et al. Updated guidelines by the European Society for the Study of Coeliac Disease. United European Gastroenterol J. 2019; 7(5): 581–2.

2- Husby S et al, AGA Clinical Practice Update on Diagnosis and Monitoring of Celiac Disease: Changing Utility of Serology and Histologic Measures: Expert Review. Gastroenterology. 2019; 156(4): 885–9.

3- Robert ME et al, Statement on Best Practices in the Use of Pathology as a Diagnostic Tool for Celiac Disease. Am J Surg Pathol 2018;42:e44-e58.

4- US Preventive Services Task Force, Bibbins-Domingo K, et al. Screening for Celiac Disease: US Preventive Services Task Force Recommendation Statement. JAMA. 2017 Mar 28;317(12):1252-1257.

5- Lazebnik LB, et al; Scientific Society of Gastroenterology of Russia. [GUIDELINES FOR DIAGNOSIS AND TREATMENT OF CELIAC DISEASE]. Eksp Klin Gastroenterol. 2015;(5):3-12.

6- Downey L, et al. Recognition, assessment, and management of coeliac disease: summary of updated NICE guidance. BMJ. 2015 Sep 2;351:h4513.

7- Nadhem O et al. Review and practice guidelines for celiac disease in 2014. Postgrad Med 2015 127(3): 259-65.

8- Hill P, et al. British Society of Gastroenterology guidelines on the diagnosis and management of coeliac disease. Gut. 2015 Apr;64(4):691-2.

9- Suomalaisen L. Duodecimin; Suomen Gastroenterologiayhdistys RY:NAsettama Työryhmä. Keliakia [Update on Current Care guidelines: coeliac disease]. Duodecim. 2011;127(3):296-7.

10- Kelly CP, et al. Advances in Diagnosis and Management of Celiac Disease. Gastroenterology 2015; 148 (6): 1175-86.

11- Trovato CM, Montuori M, Cucchiara S, Oliva S. ESPGHAN ‘biopsy-sparing’ guidelines for celiac disease in children with low antitransglutaminase during COVID-19. Eur J Gastroenterol Hepatol. 2020 Dec;32(12):1523-1526.

12- Hill ID, et al.NASPGHAN Clinical Report on the Diagnosis and Treatment of Gluten-relatedDisorders. J Pediatr Gastroenterol Nutr. 2016 Jul;63(1):156-65.

13- Jones HJ, et al. NICE clinical guideline 86. Coeliac disease: recognition and assessment of coeliac disease. Arch Dis Child. 2010 Apr;95(4):312-3.

14- Leonard MM, et al. AGA Clinical Practice Update on the Evaluation and Management of Seronegative Enteropathies: Expert Review. Gastroenterology. 2021 Jan;160(1):437-444.

15- Cheng FW, et al. Nutrition Assessment, Interventions, and Monitoring for Patients with Celiac Disease: An Evidence Analysis Center Scoping Review. J Acad Nutr Diet. 2020 Aug;120(8):1381-1406.
